# Immune evasion of multidrug-resistant bacteria: insights from lung innate immune cells and targeted therapies

**DOI:** 10.3389/fcimb.2026.1790950

**Published:** 2026-03-17

**Authors:** Yingfei Xu, Yongheng Gao, Yujuan Li, Lei Pan

**Affiliations:** Department of Pulmonary and Critical Care Medicine, Tangdu Hospital, Air Force Medical University, Xi’an, China

**Keywords:** immune escape, immunotherapy, innate immune cells, multidrug-resistant bacteria, pneumonia

## Abstract

Pneumonia is a common acute infectious disease affecting the lower respiratory tract. Innate immune cells within the lungs play a crucial role in the onset and progression of pneumonia. Various multidrug-resistant bacteria have evolved multiple strategies to evade innate immunity, posing significant challenges to the treatment of pneumonia. This review primarily explores the major innate immune cells in the lungs and their functions, the immune evasion mechanisms of common multidrug-resistant bacteria, and the latest research advances in targeted innate immunity-based anti-infective therapies. This review synthesizes recent advances in the interaction between the innate immune system of the lungs and multidrug-resistant bacteria. It systematically categorizes and compares the immune evasion mechanisms of multidrug-resistant bacteria across four core pathways. This aims to provide novel insights for treating pulmonary infections caused by these multidrug-resistant pathogens.

## 1 Introduction

Pneumonia ranks among the leading causes of global disease burden, posing a particularly severe threat to the health of children and older adults (The Lancet Healthy Longevity, 2021; Torres et al., 2021). The situation is further exacerbated by the increasing prevalence of multidrug-resistant bacterial infections (Murray et al., 2022). Common multidrug-resistant pathogens in pneumonia include methicillin-resistant Staphylococcus aureus (MRSA), carbapenem-resistant Acinetobacter baumannii (CRAB), and carbapenem-resistant Klebsiella pneumoniae, among others (Sader et al., 2019). Infections caused by these multidrug-resistant pathogens pose significant challenges for treatment (Thompson, 2022). Current research suggests that pathogens are not the key determinant of respiratory infections; rather, the body’s differential response to pathogens is the decisive factor in the onset and progression of pneumonia (Traber and Mizgerd, 2025).The pulmonary immune defense system plays a crucial role in pathogen clearance, control of inflammatory damage, and tissue repair (Holt et al., 2008). Multidrug-resistant bacteria evade clearance by the innate immune system through multiple unique mechanisms (Baxt et al., 2013). These immune evasion mechanisms pose significant challenges for treating pulmonary infections. There is an urgent need to gain a deeper understanding of the complex interactions between the host immune system and multidrug-resistant bacteria, in order to overcome current limitations in antibiotic therapy and to develop novel treatment strategies for multidrug-resistant bacterial pulmonary infections.

## 2 Immune cells in the innate defense system of the lungs

The human lungs inhale approximately 10,000 L of air daily, which contains various fine particulate matter. The lungs are exposed to these substances over the long term. Additionally, the lungs must defend against invading viruses, bacteria, and other respiratory pathogens. Therefore, the lungs must maintain a state of equilibrium, which relies on their innate defense system. This system includes various mucociliary clearance mechanisms, structural cells, and diverse immune cells (Ardain et al., 2020). This review focuses on alveolar macrophages, neutrophils, innate lymphoid cells, and innate T cells—key players in pulmonary innate immunity—and details their respective functions within this immune framework. Simultaneously, we focus on the characteristic pathways through which multidrug-resistant bacteria exploit these innate immune cells during their functional processes, revealing key entry points for multidrug-resistant bacteria to evade innate immune clearance.

### 2.1 Alveolar macrophages

Alveolar macrophages reside within the alveolar spaces and constitute the most abundant innate immune cells in the distal lung parenchyma (Shi et al., 2021). As the first cells to encounter invading pathogens and pollutants, they play critical roles in maintaining homeostasis, host defense, clearance of surfactant and cellular debris, pathogen recognition, initiation and resolution of pulmonary inflammation, and repair of damaged tissue (Allard et al., 2018; Cheng et al., 2021).

When the body is invaded by pathogens, alveolar macrophages recognize damage-associated molecular patterns (DAMPs) or pathogen-associated molecular patterns (PAMPs) (Chen et al., 2025), detecting the presence of damage byproducts or pathogens. They then continuously capture, phagocytose, sequester, and neutralize large numbers of inhaled pathogens and particles without activating the adaptive immune system, thereby protecting the fragile alveolar structure. When phagocytic macrophages engulf pathogens, they first form phagosomes through membrane invagination, which subsequently fuse with lysosomes to create phagolysosomes. The pathogens are then degraded by the hydrolytic enzymes within the lysosomes. Pathogens can also activate transcription factor EB (TFEB) within alveolar macrophages, directly regulating lysosomal production and function to accelerate pathogen elimination (Gray et al., 2016). TFEB also regulates the expression of the immune response gene 1 (IRG1) to produce itaconic acid, which inhibits key enzymes in pathogen metabolism, thereby limiting pathogen proliferation. It can also modulate multiple signaling pathways to suppress excessive inflammatory responses (Schuster et al., 2022). Through phagocytic receptors such as MARCO and MerTK, as well as receptors for apoptotic cells, alveolar macrophages efficiently phagocytose apoptotic granulocytes and epithelial cells, clearing cellular debris and extracellular matrix fragments deposited on alveolar walls. This efferocytosis process prevents dead cells from triggering proinflammatory reactions or immune responses within the alveoli, thereby exerting anti-inflammatory effects in the alveolar space (Guttenberg et al., 2023; Zhou et al., 2024). Furthermore, efferocytosis promotes the secretion of anti-inflammatory factors such as TGF-β, prostaglandin E2 (PGE2), and platelet-activating factor (PAF) by alveolar macrophages, which further suppresses inflammatory responses (Medeiros et al., 2009). Alveolar macrophages can also promote the differentiation of regulatory T cells (Tregs) under the influence of TGF-β and retinoic acid, further controlling inflammation (Kielbassa et al., 2019). Alveolar macrophages maintain a low inflammatory immune baseline within the alveolar space through the aforementioned mechanisms. This characteristic creates a low-immune-pressure microenvironment conducive to the initial colonization of multidrug-resistant bacteria. Concurrently, both the intracellular microenvironment and functional plasticity of alveolar macrophages are considered key factors enabling pathogens to evade immune surveillance. The specific mechanisms involve the following. ① Certain drug-resistant bacteria can survive within alveolar macrophages, thereby persisting in the lower respiratory tract (Distel et al., 2023). ② Following phagocytosis, alveolar macrophages produce PGE_2_ to exert anti-inflammatory effects. Concurrently, PGE_2_ inhibits natural killer cell activity by elevating intracellular cyclic adenosine monophosphate (cAMP) and downregulates MHC class II molecule expression on dendritic cell surfaces to reduce antigen presentation (Kim et al., 2019). PGE_2_ also inhibits NADPH oxidase activity in alveolar macrophages, thereby diminishing their bactericidal capacity (Das, 2018).

When phagocytic function becomes overloaded, alveolar macrophages initiate an inflammatory response by producing chemokines and cytokines (such as type I interferon, TNF-α, and IL-1β) to recruit and activate neutrophils, monocytes, and dendritic cells, thereby leading to the development of lung injury (Aegerter et al., 2022). Additionally, alveolar macrophages participate in inflammation resolution by secreting immunomodulatory cytokines such as TGF-β, IL-1Ra, and prostaglandins. During acute infection, transient alveolar macrophage depletion occurs, a phenomenon that facilitates the initiation of an effective inflammatory response. Following this depletion, the body rapidly replenishes macrophages in the alveolar microenvironment through local proliferation of residual alveolar macrophages and recruitment of circulating monocytes, thereby maintaining pulmonary function and gas exchange. Compared with resident macrophages, recruited alveolar macrophages exhibit bidirectional functional alterations: on one hand, they may produce increased IL-6 to combat bacterial infections (Aegerter et al., 2020; Mata et al., 2021), overexpress arginase 1 to enhance resistance against helminth infections (Chen et al., 2022); and limit Type 2 inflammation in house dust mite-induced allergic asthma (Machiels et al., 2017). Conversely, in severe COVID-19, recruited alveolar macrophages massively produce cytokines and chemokines such as IL-1, IL-6, and TNF-α, potentially causing lung injury and triggering systemic symptoms resembling cytokine release syndrome (Grant et al., 2021).

After inflammation is controlled, alveolar macrophages promote epithelial proliferation and collagen synthesis by metabolizing arginine into ornithine. They counteract infection-induced epithelial damage through growth factor secretion and also participate in lung tissue repair by secreting large amounts of amphiregulin. Amphiregulin, a ligand for the epidermal growth factor receptor (EGFR), collaborates with other growth factors to ensure efficient repair processes (Zaiss et al., 2015).

### 2.2 Neutrophils

Neutrophils are the most abundant type of white blood cells in peripheral blood and serve as the core effector cells in the pulmonary immune defense system. The most prominent feature of neutrophils is their phagocytic clearance of necrotic tissue debris and pathogens. Beyond directly engulfing bacteria and fungi, neutrophils employ multiple synergistic mechanisms to eliminate pathogens, including reactive oxygen species (ROS) derived from NADPH oxidase, cytotoxic granule components, and neutrophil extracellular traps (NETs). Additionally, neutrophils possess the ability to regulate the functions of other immune cells (Burn and Foti, 2021). However, both its chemotaxis migration process and intracellular bactericidal pathways have become critical targets for multidrug-resistant bacteria to achieve immune evasion.

Under homeostatic conditions, the majority of neutrophils reside in the pulmonary circulation. When alveolar macrophages recognize respiratory infections and signal accordingly, neutrophils within the vasculature rapidly migrate into the alveolar space. Neutrophils recognize these chemokine gradients through their chemokine receptors, enabling directed migration toward the site of infection. Among these chemokines, CXCL8 (IL-8) is one of the most critical mediators. CXCL8 induces neutrophil migration from the blood to the infection site by binding to CXCR1 and CXCR2 receptors on the neutrophil surface (Cambier et al., 2023). Beyond chemokines, other substances such as inflammatory lipids, serum proteins, and bacterial components also form corresponding concentration gradients, thereby regulating neutrophil migration. Neutrophils also express glycosylated RNA molecules on their surface. These glycosylated RNAs interact with P-selectin on endothelial cells, a process critical for the rapid and efficient migration of neutrophils into the alveolar space. This intricate process is susceptible to disruption by multidrug-resistant bacteria, preventing their recruitment to the site of infection. The mechanisms involved include the production of chemokine-inhibiting proteins that block neutrophil recognition and response to chemokines and direct suppression of IL-8 production by epithelial cells, thereby impairing the formation of chemokine concentration gradients (Souche et al., 2023).

The initiation of neutrophil phagocytosis depends on microbial opsonization, whereby microorganisms are coated by opsonins and subsequently recognized by specific receptors on the neutrophil surface. Complement components and immunoglobulins are the primary substances in serum that enable efficient opsonization. Once neutrophils migrate to the site of infection, opsonized pathogens can be taken up into intracellular vesicles via receptor-mediated pathways (i.e., phagocytosis). Similar to other phagocytes such as macrophages, neutrophils express multiple receptors, including pattern recognition receptors (PRRs), G protein-coupled receptors (GPCRs), and opsonin receptors. These receptors recognize microbe-associated molecular patterns (MAMPs) or host proteins involved in microbial opsonization (e.g., IgG and complement) (van Kessel et al., 2014). Multidrug-resistant bacteria can inhibit phagocytosis initiation at its source by degrading conditioning substances and blocking phagocytic recognition receptors (González-Alsina et al., 2023).

Following phagocytosis of bacteria, neutrophils generate large amounts of reactive oxygen species (ROS) through the NADPH oxidase system, including superoxide anion (O_2_^-^), hydrogen peroxide (H_2_O_2_), and hydroxyl radical (·HO). These ROS effectively destroy bacterial cell walls and nucleic acids, thereby exerting their bactericidal effects (Nguyen et al., 2017). Following phagocytosis initiation, neutrophil degranulation is activated. Neutrophils release granules to assist in eliminating invading pathogens, playing a crucial role in immune defense. Neutrophils contain four types of granules: azurophilic granules (primary granules), specific granules (secondary granules), gelatinase granules (tertiary granules), and secretory vesicles. Each granule type possesses distinct protein compositions and functions. Following initial contact between neutrophils and endothelial cells, secretory vesicles are released via exocytosis, followed sequentially by gelatinase granules, azurophilic granules, and specific granules, thereby initiating a cascade of antimicrobial activities (Teng et al., 2017). Neutrophils also exert bactericidal effects by releasing NETs, a mesh-like structure composed of DNA/histones embedded with antimicrobial peptides and enzymes derived from granules. This is considered the third effector mechanism of neutrophils (Brinkmann et al., 2004; King and Dousha, 2024). The core bactericidal pathways of neutrophils can also be specifically inhibited by multidrug-resistant bacteria, enabling immune evasion. Multidrug-resistant bacteria can suppress the bactericidal activity of neutrophil serine proteases by secreting proteases (Birnberg-Weiss et al., 2025). Multidrug-resistant bacteria have also evolved specific escape strategies against NETs, either by degrading the structural framework or antimicrobial components of NETs (Castro et al., 2025) or by directly inhibiting neutrophil NET formation (Kamoshida et al., 2018), thereby evading killing and disseminating to the lungs. Concurrently, NETs, proteases, and ROS all contribute to lung injury. Their presence is associated with worsened pulmonary damage in both animal models of pneumonia and human patients. Multidrug-resistant bacteria exploit the tissue injury microenvironment created by impaired neutrophil function or uncontrolled release of effector molecules to achieve immune evasion.

### 2.3 Innate lymphoid cells

Innate lymphoid cells (ILCs) constitute a class of innate immune cells lacking specific antigen receptors but capable of producing multiple cytokines and performing diverse functions within barrier tissues. ILCs not only defend against pathogen infections but also promote tissue repair post-infection; when their function becomes dysregulated, they may also contribute to disease pathogenesis. All ILCs share two defining characteristics: (i) absence of antigen receptor rearrangement dependent on the Recombination Activation Gene (RAG); (ii) non-expression of surface markers associated with other lymphoid and myeloid cell lineages (Stehle et al., 2018). Based on surface markers, cytokine production patterns, and required transcription factors, ILCs are classified into three types: Type 1 Lymphoid Immune Cells (ILC1s), which include the classically recognized natural killer (NK) cells. These cells primarily mediate innate Type 1 immune responses and are closely associated with antiviral and antitumor immunity; Type 2 Innate Lymphoid Cells (ILC2s), functionally similar to CD4^+^ Th2 cells, form the core of innate type 2 immune responses and participate in allergic reactions and anti-parasitic infections; Type 3 Innate Lymphoid Cells (ILC3s), functionally similar to CD4^+^ Th17 cells and Th22 cells, participate in type 3 immune responses against bacterial and fungal infections (Ardain et al., 2019).

Innate lymphoid cells lack specific antigen receptors and initiate responses by recognizing DAMPs, PAMPs, and cytokines. During pulmonary infection, NK cells and ILC3s are the most prominent cells within the innate lymphoid cell population. Upon activation, NK cells induce apoptosis in virus-infected cells by producing granzyme, perforin, TRAIL, and/or FasL, thereby playing a central role in pathogen clearance. Additionally, NK cells enhance subsequent immune responses by secreting inflammatory cytokines such as TNF-α and IFN-γ (Theresine et al., 2020). ILC3s promote the expression of IL-22 and IL-17 in response to IL-1β and IL-23 produced by myeloid cells or granulocytes, as well as through synergistic interactions with other inflammatory mediators such as PGE2 and TL1A. Beyond cytokine stimulation, activation of the ILC3 receptor NKp44 (which binds platelet-derived growth factor) induces a proinflammatory phenotype and TNF release. IL-17 mediates alveolar neutrophil recruitment and enhances neutrophil phagocytic activity, playing a crucial role in controlling several major bacterial pneumonias (Yang et al., 2021). IL-22 promotes pulmonary epithelial cell proliferation and enhances *in vitro* killing activity against Klebsiella pneumoniae by upregulating lipocalin-2 expression (Aujla et al., 2008). Potential interactions between IL-22 and IL-17 may also exist, jointly regulating pulmonary inflammatory responses.

The IL-17/IL-22 signaling pathway mediated by ILC3s, NK cell-mediated killing functions, and the activation regulatory properties of ILCs can all be targeted and disrupted by multidrug-resistant bacteria to weaken the pulmonary antimicrobial immune response. Among these, multidrug-resistant bacteria induce dysfunction of the ILC3-IL-17 signaling axis, playing a crucial role in the course of persistent multidrug-resistant bacterial infection. Multidrug-resistant bacteria may directly suppress ILC3 activation and IL-17 secretion, preventing the initiation of effective neutrophil-mediated antimicrobial immunity (Hoffmann et al., 2021) or abnormally activate the ILC3-IL-17 signaling axis, triggering pathological tissue damage. They may also inhibit downstream antimicrobial signaling of IL-17A, causing IL-17A to mediate tissue damage while completely losing its antimicrobial function (Su et al., 2025; Zhang et al., 2025), thereby creating a favorable pathological microenvironment for persistent bacterial infection.

### 2.4 Innate T cells

Innate T cells (ITCs), including invariant natural killer T (iNKT) cells, mucosa-associated invariant T (MAIT) cells, and γδ T cells, are tissue-resident unconventional T cells possessing characteristics of both innate and adaptive immunity. Research on the mechanisms of innate T cells in pulmonary infections remains limited (Gao and Williams, 2015). Innate T cells express a limited repertoire of T-cell receptors (TCRs) and can be activated via PRRs or cytokines in an antigen-independent manner. Multiple types of innate T cells can be recruited from the circulation to infected lung tissue within hours during Streptococcus pneumoniae pneumonia, where they accumulate in large numbers.

Innate-like natural killer T (iNKT) cells express αβTCRs and recognize glycolipid antigens presented by the MHC-I-like molecule CD1d. In Streptococcus pneumoniae pneumonia, iNKT cells are activated in the lung via conventional type 1 dendritic cells (cDC1s) and play a crucial role in pulmonary defense (Murray et al., 2022). Research indicates that during multidrug-resistant bacterial infections, the activation of iNKT cells significantly enhances the phagocytic and cytotoxic capabilities of macrophages against these pathogens. Conversely, certain bacteria can evade the immune system by impairing the antigen-presenting function of the host cell’s CD1d molecule, thereby suppressing iNKT cell activation (Chandra et al., 2018). Mucosa-associated invariant T (MAIT) cells express restricted αβTCRs that recognize riboflavin metabolites presented by the MHC-I-related receptor MR1. MR1-mediated antigen presentation activates MAIT cells to produce inflammatory cytokines or eliminate infected cells via granzyme production and apoptosis induction (Hartmann et al., 2018). MAIT cells also play a critical role in respiratory viral infections. However, excessive MAIT cell responses may lead to dysregulated immune responses and poor outcomes. Research indicates that multidrug-resistant bacteria can evade antigen recognition by MAIT cells by downregulating the riboflavin synthesis pathway (Preciado-Llanes et al., 2020). γδ T cells express γδ TCRs with limited diversity and typically lack CD4 or CD8 molecules. In pulmonary infections, γδ T cells primarily mediate early responses to pathogens. Activated γδ T cells produce IL-17A and IFN-γ, promoting the recruitment of effector cells (e.g., macrophages and neutrophils), granuloma formation, and Th17 immune responses (Peters et al., 2018; Omar et al., 2020).

**Figure 1:**
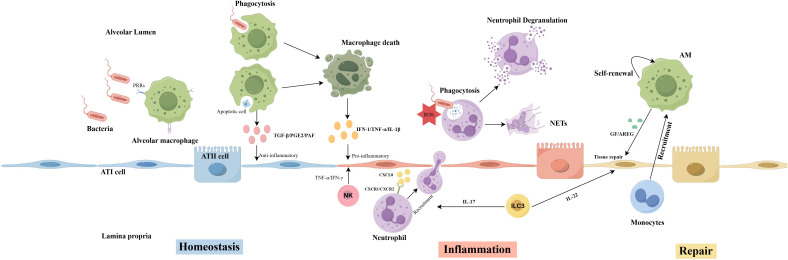
Mechanisms of innate immune cells in the lung during homeostasis, inflammation, and repair. Homeostasis: Alveolar macrophages maintain alveolar epithelial cell homeostasis by directly phagocytosing bacteria and secreting anti-inflammatory factors. Inflammation: Alveolar macrophages and NK cells promote the transition of alveolar epithelial cells to an inflammatory state by secreting pro-inflammatory factors, recruiting neutrophils for further bacterial clearance. ILC3s mediate neutrophil recruitment and enhance neutrophil phagocytic activity by secreting IL-17. Repair: Alveolar macrophages and ILC3s also play crucial roles in the tissue repair following the resolution of inflammation.

In summary, innate immune cells play a complex, multilayered role in maintaining pulmonary homeostasis, regulating inflammatory responses, and facilitating tissue repair. They eliminate pathogens through various mechanisms, preventing the onset and progression of pneumonia (**Figure 1**).

## 3 Immune evasion mechanisms of multidrug-resistant bacteria

In pulmonary infections, an increasing number of multidrug-resistant bacteria are being detected, posing significant challenges to treatment. This article will focus on four core pathways: phagosome–lysosome maturation, complement activation and opsonophagocytosis, inflammasome signaling and immune paralysis mechanisms, and metabolic reprogramming of immune cells. It will specifically highlight the evasion mechanisms employed by the four most common and characteristic multidrug-resistant pathogens in pulmonary infections—Klebsiella pneumoniae, Pseudomonas aeruginosa, Acinetobacter baumannii, and Staphylococcus aureus—when confronting the body’s innate immune defenses. This analysis aims to provide insights for developing targeted therapeutic strategies.

### 3.1 Pseudomonas aeruginosa

Regarding interference with phagosome–lysosome maturation, Pseudomonas aeruginosa’s *nirD* gene (a nitrite reductase gene) utilizes nitric oxide produced by inducible nitric oxide synthase in neutrophils to reduce nitrite to ammonia. This inhibits phagosome maturation, ultimately enabling escape from neutrophil killing (Nakatsuka et al., 2023).

Regarding interference with complement activation and opsonophagocytosis, P. aeruginosa employs multiple surface structures and secreted proteins to achieve escape. During chronic infections, the bacterium reduces immunogenicity by shedding flagella, thereby evading phagocytic attacks. Fimbriae promote bacterial aggregation, facilitating microcolony and biofilm formation. Biofilms consist of bacterial cells enveloped by extracellular polysaccharides such as alginate, Pel polysaccharide, and Psl polysaccharide. This coating can shield lipopolysaccharide (LPS) from immune recognition, reduce complement activation, and minimize complement deposition on the bacterial surface (Haidar et al., 2024). Lipopolysaccharide (LPS), a major component of P. aeruginosa’s outer membrane, is a macromolecular complex composed of lipoteichoic acid, core polysaccharide, and O-antigen polysaccharide. P. aeruginosa evades host immune attacks by modifying the structure of Lipid A (Cigana et al., 2009). The O-antigen in LPS blocks direct contact between complement and bacterial surface target molecules. Additionally, O-antigen-specific IgG_2_ antibodies shield the O-antigen, preventing complement components from contacting the bacterial membrane and aiding resistance to complement system attacks (Pham et al., 2021). Furthermore, P. aeruginosa secretes elastase and phospholipase through its type II secretion system. Both enzymes degrade pulmonary surfactant, which has conditioning effects (Kuang et al., 2011), thereby evading phagocytosis by immune cells. Phospholipase also inhibits the oxidative burst response of neutrophils, resisting reactive oxygen species-mediated killing. Secreting alkaline proteases via the Type I secretion system degrades host complement, reducing phagocytic efficiency of leukocytes. It also degrades flagellar proteins to evade TLR5 recognition of the bacterial cell body (Yang et al., 2017).

In disrupting inflammasome signaling and inducing immune paralysis, P. aeruginosa participates in immune evasion through quorum sensing mechanisms. The acyl-cysteinolactones it produces act as autoinducers for interbacterial signaling, influencing biofilm formation (De Kievit, 2009; Touati et al., 2025). Certain acyl-cysteinolactones also directly target macrophages, inhibiting LPS-induced NF-κB activation and inflammatory cytokine production, thereby creating favorable conditions for bacterial survival (Zhang et al., 2014). Following uptake by host cells, Exotoxin A inhibits the translation elongation factor EF2, disrupting protein synthesis and potentially suppressing the host immune response. Pyocyanin induces neutrophil apoptosis in a dose-dependent manner, hindering bacterial clearance (Managò et al., 2015).

Regarding immune cell metabolic reprogramming, P. aeruginosa quorum sensing molecules directly modulate macrophage function, influencing their metabolic polarization (Chakraborty et al., 2024). Local hypoxia induced by biofilm formation triggers glycolytic adaptation in immune cells via HIF-1α, a mechanism potentially exploited by bacteria to evade immune clearance (Hayek et al., 2021).

### 3.2 Klebsiella pneumoniae

In disrupting phagosome–lysosome maturation, Klebsiella pneumoniae can persist within specialized vesicles in macrophages (termed Klebsiella pneumoniae-containing vesicles, KCVs). It achieves this by activating the PI3K-AKT-Rab14 pathway, preventing KCV fusion with lysosomes and thereby evading destruction by lysosomal hydrolases (Cano et al., 2015).

Regarding interference with complement activation and opsonophagocytosis, K. pneumoniae’s capsular polysaccharide (CPS) serves as a core virulence factor. The capsule acts as a physical barrier, preventing complement binding and deposition while inhibiting the membrane attack complex C9 pore from interacting with the bacterial membrane (Doorduijn et al., 2016). The capsules of K1 and K2 strains lack mannose, thereby inhibiting lectin-mediated phagocytosis and subsequent proinflammatory signaling (Xu et al., 2024). Sialic acid in the capsular polysaccharide mimics host cells by binding to the major histocompatibility complex class I receptors on neutrophil surfaces, aiding bacterial evasion of immune responses or repelling neutrophils (Opal, 2014). High expression of fructose-1,6-bisphosphate aldolase on the surface of K1-type highly virulent K. pneumoniae protects the bacteria from phagocytosis and killing by neutrophils in high-sugar environments (Lee et al., 2020). Paradoxically, capsular deficiency sometimes enhances resistance. For instance, strains with inactivated *wcaJ* genes exhibit increased C3b complement deposition yet show enhanced resistance to immune killing, potentially due to failed complement activation (Bain et al., 2024). Capsular polysaccharides also interfere with dendritic cell activation and maturation, impairing antigen presentation, reducing Th1 cytokine and TLR4 expression, and decreasing natural killer cell migration to infection sites (Nicolò et al., 2022). Multiple outer membrane proteins on K. pneumoniae surfaces contribute to evasion; for example, downregulation of OmpK36 reduces exposure of surface immune epitopes, lowering recognition by immune cells (Tsai et al., 2011). Overexpression of AraC family transcription factors (e.g., RamA) further modifies the lipid A structure of LPS, thereby reducing macrophage phagocytosis and clearance (De Majumdar et al., 2015).

K. pneumoniae suppresses inflammatory signaling through multiple mechanisms to interfere with inflammasome signaling and induce immune paralysis. The capsule masks LPS, attenuates TLR4 signaling, and reduces proinflammatory cytokine production (Wu et al., 2009). The outer membrane protein OmpA inhibits proinflammatory signaling activation in airway epithelial cells, reducing immune cell recruitment (March et al., 2011). K. pneumoniae also directly suppresses NF-κB function by interfering with posttranslational modifications (e.g., ubiquitination, SUMOylation, and NEDDylation), such as upregulating the deubiquitinating enzyme CYLD, affecting the de-SUMOylating enzyme SENP2, interfering with the de-NEDDylating enzyme CSN5, and inactivating the NEDD8-binding protein Ubc12, thereby suppressing NF-κB-dependent inflammation. It also upregulates MAPK phosphatase 1 (MKP-1), reducing IL-8 production in epithelial cells by inhibiting MAPK phosphorylation. In macrophages, it induces type I interferon production, upregulates miRNA let-7 expression to reduce SUMOylation levels, and simultaneously induces expression of the innate immune protein SARM1. This blocks activation of the NF-κB and IRF3 pathways, decreases MAPK phosphorylation, and inhibits NLRP3 and AIM2 inflammasome activation (Sá-Pessoa et al., 2020).

Regarding immune cell metabolic reprogramming, K. pneumoniae activates host TLR2/TLR4 signaling via its capsular polysaccharide. Under the action of transcription factor STAT6, it induces a unique polarization state (M-Kp) in pulmonary macrophages characterized by glycolytic metabolism. This state provides an intracellular survival microenvironment for the bacterium, aiding its evasion of clearance (Dumigan et al., 2022). Furthermore, K. pneumoniae reduces phosphatidylserine expression on neutrophil surfaces, converting normal neutrophil apoptosis to necrotic death, and induces the generation of a low-activity neutrophil subset, all of which favor bacterial survival (Jondle et al., 2018).

### 3.3 Acinetobacter baumannii

In disrupting phagosome–lysosome maturation, A. baumannii survives within macrophages after phagocytosis by producing catalase to counteract the bactericidal effects of reactive oxygen species (Sato et al., 2019).

Regarding interference with complement activation and opsonophagocytosis, A. baumannii modulates the complement pathway through multiple mechanisms. Its outer membrane protein A (OmpA) mediates bacterial adhesion by binding to fibronectin on host cell surfaces and is associated with biofilm formation (Jin et al., 2011; Tiku et al., 2021). A. baumannii recruits complement factor H to its surface, thereby evading complement system activity (Kim et al., 2009). Its plasminogen-like protein CipA promotes C3b cleavage and fibrin network degradation by inhibiting the alternative complement pathway (Ries et al., 2022). Capsular polysaccharides, lipopolysaccharides, and OmpA also contribute to resistance against complement-mediated killing. Furthermore, A. baumannii suppresses neutrophil extracellular trap (NET) formation, such as by inhibiting CD11a expression on neutrophil surfaces to block pathogen-mediated neutrophil adhesion (Kamoshida et al., 2018). A. baumannii can also directly adhere to neutrophil surfaces, enhance neutrophil chemotaxis by increasing IL-8 production, and be transported alongside invading neutrophils to facilitate infection spread (Kamoshida et al., 2016).

Regarding interference with inflammasome signaling and induction of immune paralysis, the capsular polysaccharide of A. baumannii suppresses its activation of proinflammatory cytokines, reducing innate immune cell recruitment and thereby evading clearance (Kim et al., 2009; Ries et al., 2022). Research indicates that certain A. baumannii strains enhance neutrophil chemotaxis by increasing IL-8 production, and this modulation of chemokines may contribute to immune paralysis (Kamoshida et al., 2016).

### 3.4 Staphylococcus aureus

In disrupting phagosome–lysosome maturation, Staphylococcus aureus employs multiple mechanisms to resist intracellular killing after phagocytosis. The sorting enzyme-anchoring protein AdsA it expresses catalyzes the dephosphorylation of adenosine monophosphate, diphosphate, and triphosphate, increasing extracellular adenosine concentrations during infection and promoting S. aureus survival within neutrophils (Thammavongsa et al., 2009). The peroxidase inhibitor secreted by S. aureus specifically binds to and inhibits myeloperoxidase (MPO), thereby blocking neutrophil killing activity (Herdendorf et al., 2024). Additionally, three extracellular adhesion proteins produced by S. aureus—Eap, EapH1, and EapH2—inhibit neutrophil serine proteases (elastase, cathepsin G, and protease 3), thereby promoting bacterial survival (Stapels et al., 2016).

Regarding interference with complement activation and opsonophagocytosis, S. aureus prevents the membrane attack complex from contacting its cell membrane via a thick peptidoglycan layer, whereas capsular components also contribute to resistance against neutrophil phagocytosis. S. aureus secretes multiple proteins that interfere with complement activation and phagocytosis: Protein A (SpA), Staphylococcal Complement Inhibitor (SCIN), Extracellular Fibrinogen Binding Protein (Efb), and its homolog Ecb (Extracellular Complement Binding Protein), Staphylococcal Immunoglobulin Binding Protein (Sbi), and surface protein SdrE all interfere with complement activation and its mediated phagocytosis. For example, SdrE captures complement regulatory factor H, inhibiting the complement bypass pathway (Paiva et al., 2023). S. aureus also secretes staphylococcal superantigen-like proteins (SSLs), phenol-soluble modulators (PSMs), chemotaxis inhibitory proteins (CHIPS), and formyl peptide receptor-like 1 inhibitor (FLIPr), which interfere with neutrophil extravasation and chemotaxis. For instance, SSL5 and SSL11 bind to PSGL-1 on leukocyte surfaces, disrupting neutrophil binding to P-selectin and adhesion/rolling processes (Chen et al., 2019). S. aureus enterotoxin-like toxin X (SElX) binds neutrophils via surface receptors, inhibiting phagocytosis (Tuffs et al., 2017). Furthermore, S. aureus nuclease (Nuc) degrades NETs, rendering them incapable of bactericidal activity. Nucleotides released during degradation are converted to deoxyadenosine by AdsA, triggering macrophage apoptosis and preventing phagocytes from entering the abscess core (Thammavongsa et al., 2013).

To disrupt inflammasome signaling and induce immune paralysis, S. aureus secretes multiple two-component leukotoxins such as PVL, γ-hemolysin AB and CB (HlgAB, HlgCB), and LukAB. These toxins activate the NLRP3 inflammasome pathway through pore formation and potassium efflux, inducing cell death in immune cells like macrophages and neutrophils to evade immune clearance (Spaan et al., 2017). Additionally, phenol-soluble modulators (PSMs) and α-hemolysin (Hla) can induce necrosis in innate immune cells, thereby regulating innate immune responses (Berube and Bubeck Wardenburg, 2013; Surewaard et al., 2013).

In summary, common multidrug-resistant bacteria have evolved multiple mechanisms to evade clearance by the innate immune system. Although these mechanisms exhibit significant species specificity, they remain concentrated within four core components of the pulmonary innate immune system. A cross-species summary comparison of these mechanisms is detailed in **Table 1**. Concurrently, three Gram-negative pathogens—Pseudomonas aeruginosa, Klebsiella pneumoniae, and Acinetobacter baumannii—share common mechanisms, such as ① relying on outer membrane structures to form physical barriers that prevent complement deposition and phagocytic recognition and ② modulating TLR4 recognition of LPS through diverse mechanisms (LPS modification, capsular shielding, biofilm masking). Staphylococcus aureus, however, heavily relies on secreted toxins to directly kill immune cells and employs surface and secreted proteins to interfere with complement, chemotaxis, and phagocytosis through multiple targets. Notably, some immune evasion mechanisms in multidrug-resistant bacteria represent universal virulence traits, whereas others are key factors in resistance development itself, such as the following: ① biofilms—serving as both physical barriers against immune cells and barriers limiting antibiotic penetration, making them core factors in resistance (Chen et al., 2024); ② efflux pumps and modifying enzymes—transcription factors like RamA in Klebsiella pneumoniae regulate both capsular formation and efflux pumps in multidrug-resistant bacteria (Xu et al., 2021). The targeted disruption of pulmonary innate immunity by multidrug-resistant bacteria renders the lungs’ first line of defense ineffective, leading to difficulties in eliminating multidrug-resistant bacteria, exacerbating infections, and increasing their prevalence in human populations.

**Table 1:** Summary of immune evasion strategies of major MDR bacteria.

Immune evasion strategies	Common characteristics	Specific characteristics
Phagosome–lysosome maturation	Block fusion between phagosomes and lysosomes; resist lysosomal proteolytic enzymes and reactive oxygen species-mediated killing	P. aeruginosa: *nirD*: utilizes host NO to reduce nitrite to ammonia, inhibiting phagosome maturation. Phospholipase: inhibits neutrophil oxidative burst and resists reactive oxygen species-mediated cytotoxicity.
		K. pneumoniae: PI3K-AKT-Rab14 pathway: inhibits fusion of KCV with lysosomes.
		A. baumannii: Catalase: resists macrophage-mediated reactive oxygen species killing.
		S. aureus: AdsA catalyzes adenosine synthesis to promote intracellular survival in phagocytes; Eap family: inhibits neutrophil serine proteases; peroxidase inhibitor: suppresses MPO.
Complement activation and opsonophagocytosis	Physical barriers prevent complement deposition; recruit complement regulators; secrete proteases to degrade complement components	P. aeruginosa: flagellar loss: loss of flagella during chronic infection reduces immunogenicity and evades phagocytic attacks; Fimbriae: promote aggregation to form microcolonies; biofilm: envelopes cells, inhibits complement deposition, shields LPS; LPS modification: alters lipid A; O antigen blocks complement binding; elastase: degrades pulmonary surfactant; alkaline protease: degrades complement; degrades flagellar proteins to evade TLR5 recognition; phospholipase: degrades pulmonary surfactant and suppresses neutrophil oxidative burst, resisting reactive oxygen species-mediated killing.
		K. pneumoniae: *wcaJ* inactivation: Increases complement deposition but failed activation, enhancing resistance to immune killing; CPS: physical barrier blocking complement; lack of mannose blocking the complement-mucin pathway; sialic acid mimicking host; fructose aldolase inhibiting phagocytosis; outer membrane protein: OmpK36 downregulation reducing epitope exposure; LPS modification: RamA modifies LPS to reduce macrophage uptake.
		A. baumannii: complement factor H recruitment: recruits host regulatory proteins to evade complement attack; CipA protein: inhibits the alternative complement pathway and degrades fibrin; CPS: inhibits bacterial-host interactions, suppresses phagocytosis, reduces immune recruitment; OmpA protein: mediates adhesion to pulmonary epithelial cells, evades phagocytosis; suppresses NETs: inhibits CD11a expression, blocks neutrophil adhesion.
		S. aureus: physical barriers: thick peptidoglycan layer impedes MAC; capsule resists phagocytosis; complement-interfering proteins: SCIN, Efb/Ecb, Sbi, SdrE, SpA (binds antibody Fc); chemotaxis interference: SSL5/SSL11 inhibit adhesion; CHIPS/FLIPr inhibit chemotaxis; PSMs regulate immunity; SElX: binds neutrophils, directly inhibits phagocytosis; Nuclease (Nuc): degrades NETs.
Inflammasome signaling versus immune paralysis	Induce immune cell death; suppress pro-inflammatory signaling and inflammasome activation	P. aeruginosa: quorum sensing: acyl-cysteinolactones directly act on macrophages, inhibiting NF-κB and inflammatory factors; exotoxin A: inhibits EF2, blocks protein synthesis, suppresses immune response; pyocyanin: induces neutrophil apoptosis.
		K. pneumoniae: capsule masks LPS: weakens TLR4 signaling, reduces proinflammatory cytokine production; OmpA inhibits proinflammatory signaling, decreases immune cell recruitment; posttranslational modifications: regulates CYLD, SENP2, CSN5, Ubc12, suppresses NF-κB.
		A. baumannii: IL-8 upregulation: adheres to neutrophils and spreads via their transport; capsular reduction: suppresses proinflammatory factors, reducing immune cell recruitment.
		S. aureus: pore-forming toxins: HlgAB/CB, LukAB Activate NLRP3 to induce pyroptosis; Hla induces immune cell necrosis.
Metabolic reprogramming of immune cells	Alter host cell energy metabolism pathways; Induce abnormal phenotypic polarization of immune cells	P. aeruginosa: quorum sensing: acyl-cysteinolactones directly regulate macrophage function and metabolic polarization; indirect biofilm induction: local hypoxia induces glycolytic adaptation via HIF-1α.
		K. pneumoniae: induction of M(Kp) polarization: capsular polysaccharides activate TLR2/TLR4-STAT6, inducing glycolytic M(Kp) polarization in macrophages to provide an intracellular survival microenvironment; Induction of low-activity neutrophil subsets weakens bactericidal capacity.

## 4 New strategies for intrinsic immune therapy in the lung

### 4.1 Regulate the function of the innate immune defense system

Research indicates that certain drugs, such as vitamin D_3_, sodium phenylbutyrate (PBA), and aroylated phenylenediamine (HO53), can stimulate macrophages. Through the production of antimicrobial peptides, the generation of reactive oxygen species, and synergistic effects with antibiotics, they collectively enhance the clearance of pathogenic bacteria. Certain nutrients, such as L-valine and L-arginine, can stimulate phagocytosis in immune cells, thereby accelerating the rate at which pathogens are eliminated from the host (Chen et al., 2017). Research on treating drug-resistant mycobacteria has revealed that itaconic acid esters (e.g., dimethyl itaconate, DMI) enhance immune defense by maintaining inflammatory homeostasis, activating autophagy and promoting phagosome maturation, and downregulating STAT3 signaling (Kim et al., 2023). Mesenchymal stem cell-derived microvesicles also modulate the innate immune system by enhancing bacterial phagocytosis in monocytes and regulating the immune function of both monocytes and alveolar macrophages (Monsel et al., 2015). Notably, regulating the innate immune response requires consideration of the infection stage. For instance, during the early phase, GM-CSF antagonists help prevent activation of the inflammatory cascade (De Luca et al., 2020), whereas during recovery, inhaled GM-CSF promotes restoration of alveolar macrophage numbers and accelerates inflammation resolution (David et al., 2025). If the phasing and dosing strategy are mismatched, not only will therapeutic efficacy fail to materialize, but it may also accelerate infection progression or exacerbate tissue damage.

### 4.2 Training immunity for prevention and control of pulmonary infections

The innate immune system can also develop immunological memory when stimulated by pathogenic microorganisms, vaccines, or damage-associated molecular patterns (DAMPs). This enables a stronger response upon subsequent exposure to the same or unrelated pathogens—a process termed trained immunity. Trained immunity exerts unique effects in both the prevention and treatment of pulmonary infections. For instance, studies have demonstrated that BCG vaccination confers immunoprotection against lung infections caused by other pathogens (Giamarellos-Bourboulis et al., 2020), achieved by enhancing neutrophil recruitment and lung macrophage function in the lungs (Kang et al., 2023). Inappropriate activation of trained immunity may lead to adverse consequences, as it could promote long-term pro-inflammatory alterations in monocytes/macrophages, thereby driving the progression of chronic inflammatory diseases such as atherosclerosis (Bahrar et al., 2023). In elderly populations, BCG vaccination has been associated with an increased risk of hospitalization for arrhythmia (Dulfer et al., 2024). Conversely, an overactivated immune response may cause secondary lung injury. For instance, trained alveolar macrophages may exhibit excessive inflammatory responses upon restimulation, exacerbating pulmonary damage (Prevel et al., 2025); similarly, excessive activation of ILC2s can induce persistent eosinophilic inflammation and airway dysfunction (López et al., 2024).

### 4.3 Adoptive cellular immunotherapy

Macrophages play a crucial role in innate immunity. Recent studies have combined adoptive macrophage therapy with photodynamic therapy to achieve targeted antibacterial effects. This approach involves introducing the photosensitizer Lyso700D into the lysosomes of mouse macrophages and subsequently injecting it into the host via intravenous or combined intravenous and intraperitoneal routes. Leveraging the innate chemotaxis of macrophages, bacteria were tracked and phagocytosed into lysosomes, bringing the bacteria into close proximity with the photosensitizer. Near-infrared light irradiation of the infected site then induced the photosensitizer to generate reactive oxygen species, eliminating the bacteria (Wang et al., 2023). This strategy provides novel insights for treating multidrug-resistant bacterial infections in the lungs. Currently, the efficacy of this therapeutic approach has only been validated in animal studies, with numerous obstacles remaining for clinical translation: allogeneic macrophages infused carry the risk of immune rejection, potentially triggering excessive host immune responses and pulmonary inflammation; infused cells may non-specifically accumulate in reticuloendothelial organs such as the liver and spleen, failing to efficiently concentrate at pulmonary infection sites. This reduces therapeutic efficacy while increasing systemic inflammatory risks.

Therefore, therapies targeting the innate immune system are a double-edged sword. Their successful application relies on precise biomarker guidance, strict timing selection, and individualized assessment of the patient’s immune status.

## 5 Summary

The innate immune system in the lungs plays a crucial role in defending against pathogen invasion, with multiple immune cells performing complex functions. These cells not only directly phagocytose and eliminate pathogens but also regulate inflammatory responses and activate adaptive immune responses, forming a comprehensive defense system. However, multidrug-resistant bacteria evade clearance by the innate immune system through various mechanisms, leading to infection spread and posing significant therapeutic challenges. Targeted therapies directed at the innate immune system differ substantially from traditional antibiotic approaches. Enabling precise immunomodulation, acting synergistically with antibiotics, and personalized comprehensive treatment strategies offer new hope for managing multidrug-resistant pulmonary infections.
